# Individual Segmentation of Intertwined Apple Trees in a Row via Prompt Engineering †

**DOI:** 10.3390/s25154721

**Published:** 2025-07-31

**Authors:** Herearii Metuarea, François Laurens, Walter Guerra, Lidia Lozano, Andrea Patocchi, Shauny Van Hoye, Helin Dutagaci, Jeremy Labrosse, Pejman Rasti, David Rousseau

**Affiliations:** 1 Laboratoire Angevin de Recherche en Ingénierie des Systèmes (LARIS), Université d’Angers, 49000 Angers, France; herearii.metuarea@univ-angers.fr (H.M.); pejman.rasti@univ-angers.fr (P.R.); 2 Institut de Recherche en Horticulture et Semences, Institut national de recherche pour l’agriculture, l’alimentation et l’environnement, 49070 Beaucouzé, France; francois.laurens@inrae.fr; 3 Research Centre Laimburg, 39040 Auer, Italy; walter.guerra@laimburg.it; 4 Institut de Recerca i Tecnologia Agroalimentàries, 08140 Barcelona, Spain; lidia.lozano@irta.cat; 5 Agroscope, 8820 Waedenswil, Switzerland; andrea.patocchi@agroscope.admin.ch; 6 Better3fruit N.V., 3202 Rillaar, Belgium; shauny@better3fruit.com; 7 Department of Electrical-Electronics Engineering, Eskisehir Osmangazi University, 26480 Eskisehir, Turkey; helindutagaci@gmail.com; 8 Hiphen, 84140 Avignon, France; jlabrosse@hiphen-plant.com

**Keywords:** tree segmentation, foundation models, prompt engineering, zero-shot learning, dataset, phenotyping

## Abstract

Computer vision is of wide interest to perform the phenotyping of horticultural crops such as apple trees at high throughput. In orchards specially constructed for variety testing or breeding programs, computer vision tools should be able to extract phenotypical information form each tree separately. We focus on segmenting individual apple trees as the main task in this context. Segmenting individual apple trees in dense orchard rows is challenging because of the complexity of outdoor illumination and intertwined branches. Traditional methods rely on supervised learning, which requires a large amount of annotated data. In this study, we explore an alternative approach using prompt engineering with the Segment Anything Model and its variants in a zero-shot setting. Specifically, we first detect the trunk and then position a prompt (five points in a diamond shape) located above the detected trunk to feed to the Segment Anything Model. We evaluate our method on the apple REFPOP, a new large-scale European apple tree dataset and on another publicly available dataset. On these datasets, our trunk detector, which utilizes a trained YOLOv11 model, achieves a good detection rate of 97% based on the prompt located above the detected trunk, achieving a Dice score of 70% without training on the REFPOP dataset and 84% without training on the publicly available dataset.We demonstrate that our method equals or even outperforms purely supervised segmentation approaches or non-prompted foundation models. These results underscore the potential of foundational models guided by well-designed prompts as scalable and annotation-efficient solutions for plant segmentation in complex agricultural environments.

## 1. Introduction

In modern computer vision for plant science, the recent literature is dominated by the use of supervised or self-supervised deep learning methods [1,2,3]. Although powerful, a limitation of these approaches is the lack of generalization and the risk of overfitting the data used for training. This is especially the case for plant imaging in outdoor conditions due to the variability of lighting, the diversity of plants, and the complexity of the plant shape, which evolves with plant growth. A solution to these limitations has recently appeared with the introduction of foundation models [4,5,6] trained on an extremely large amount of data (typically 1 billion). The foundation models demonstrate very good generalization capabilities for any type of data and excellent results when they are fine-tuned or guided with few prompts. This development opens a new era in deep learning methods, where the bottleneck is no longer the annotation of images but the automation of prompt generation for the effective use of foundation models. The basic interest is that the time for annotation is considerably reduced because foundation models need only prompt engineering to compete with state-of-the-art standard supervised techniques. We follow this trend of prompt engineering for a specific task of plant imaging, apple tree segmentation [7], which has only recently been tackled with the standard deep learning approach.

**Figure 1:**
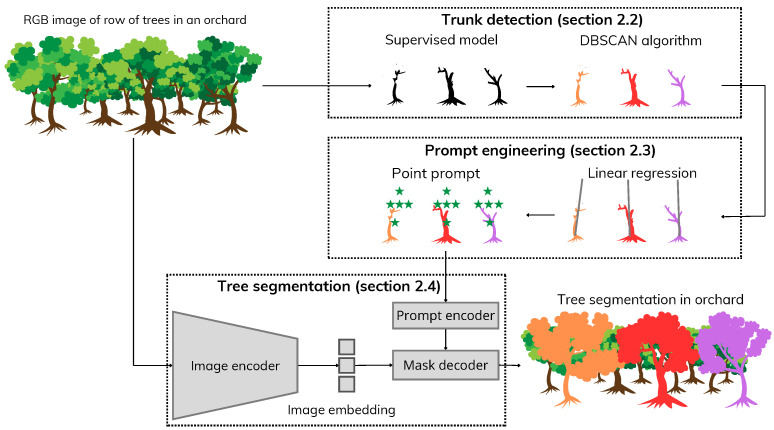
Proposed workflow of individual tree detection algorithm. First, a row of trees was photographed using an RGB camera (RGB image in orchards). In this image, an algorithm automatically segments tree trunks (trunk segmentation). Then, points (showed in green star) are defined in the space where the foliage of the tree is supposed to be, that is, above each trunk (prompt engineering). Finally, the trees are automatically segmented from the prompts and the RGB image (tree segmentation). The final result shows detected and segmented trees each marked with a different color.

We focus on the segmentation of individual apple trees in a row, in dense orchards. Such a situation is of significant importance in orchards dedicated to variety testing or breeding programs. In fact, in these orchards, each tree can correspond to a different variety and should therefore be phenotyped individually. Due to the high density of such orchards, adjacent branches may be intertwined, making tree instance segmentation a challenging task. To the best of our knowledge, we demonstrate for the first time the possibility of solving this task with a zero-shot learning approach as depicted by the prompt engineering pipeline provided in Figure 1.

The current methods [8,9,10] for individual tree separation leverage the structural information of trees in the point clouds. These methods have been extended to point clouds obtained from various sources such as Light Detection and Ranging sensors (or LIDAR sensors) [11] and stereovision techniques [9,12]. In contrast to these lines of work, La et al. [13] exploited the internal depth information in color monocular images through a fine-tuned segmentation model. Such depth cues appeared to be of sufficient quality to enable good tree segmentation [13]. More recently, visual prompting models have emerged as a new paradigm for image segmentation [14]. In this work, we explore their applications to individual tree segmentation based on color images.

Prompt engineering arises as a new paradigm with various strategies depending on the type of prompts and how they are used to boost few-shot learning [15,16,17,18,19]. Specific prompt engineering strategies have been adapted to various application domains as recently observed in medical imaging [20]. Similar approaches have been used for plant imaging [21,22,23,24,25,26,27], and the proposition of this study follows this trend. In related work on prompt engineering for plant imaging in orchards and vineyards, Torres-Lomas et al. [26] used an automatic mask generator to segment individual grapes in vineyards. More recently, Zhang et al. [27] leveraged a pre-trained ECLIP model to generate keypoint locations for single fruits using both images and text. However, these methods were developed for objects captured from neutral backgrounds. To the best of our knowledge, no previous work has applied prompt-based segmentation in orchard environments with complex and natural backgrounds, where tree trunks are partially occluded and illumination varies strongly.

The remainder of this paper is organized as follows. First, we describe the global pipeline shown in Figure 1 and the apple REFPOP dataset produced for this work. Relevant references to each subpart of the pipeline are listed to relate our work with the state-of-the-art and justify our choice. We then assess the proposed pipeline and compare it with the supervised approach of [13] on our dataset and on the dataset of [13]. Finally, we conclude the paper and discuss the implications of the results and future perspectives.

## 2. Materials and Methods

The proposed workflow comprises three main stages: trunk detection, prompt engineering, and tree segmentation. As the input, we have a RGB image of a row of trees. As the output, we obtain an instance segmentation mask. In the first stage, a supervised deep neural model detects tree trunks from an RGB image. This algorithm returns a semantic segmentation mask (Figure 1, Trunk detection). In the second stage, a regression line is fitted to each segmented trunk. Using these lines as references, a set of points is generated in the region located above the trunks, which corresponds to the expected position of the tree foliage. This step, referred to as prompt engineering (Figure 1, prompt engineering), produces prompt points that serve as input cues for the subsequent tree segmentation stage. In the final stage, a visual foundation model leverages both the RGB image and prompt points to perform tree segmentation (Figure 1, tree segmentation). The model generates a segmentation mask, in which each instance label corresponds to a distinct tree. We now describe each stage of the pipeline in detail.

### 2.1. Orchard Description, Data Acquisition and Annotation

The images in the dataset were collected from five orchards of the apple REFPOP network [28], located in Angers, France; Lleida, Spain; Laimburg, Italy; Wädenswil, Switzerland; and Rillaar, Belgium. Spanning five European countries, this network enables refined genomic and phenotypic studies of apples, with a focus on gene–environment interactions, climate response, genomic prediction, and cultivar evaluation.

The orchard layout consisted of rows of apple trees trained in a vertical trellis structure with support poles. The trees were spaced 87 cm to 1 m apart, with an average height ranging from 1 to 3 m. The trees were eight years old. The distance between the rows varied from 2.5 to 4 m, depending on the site.

**Figure 2:**
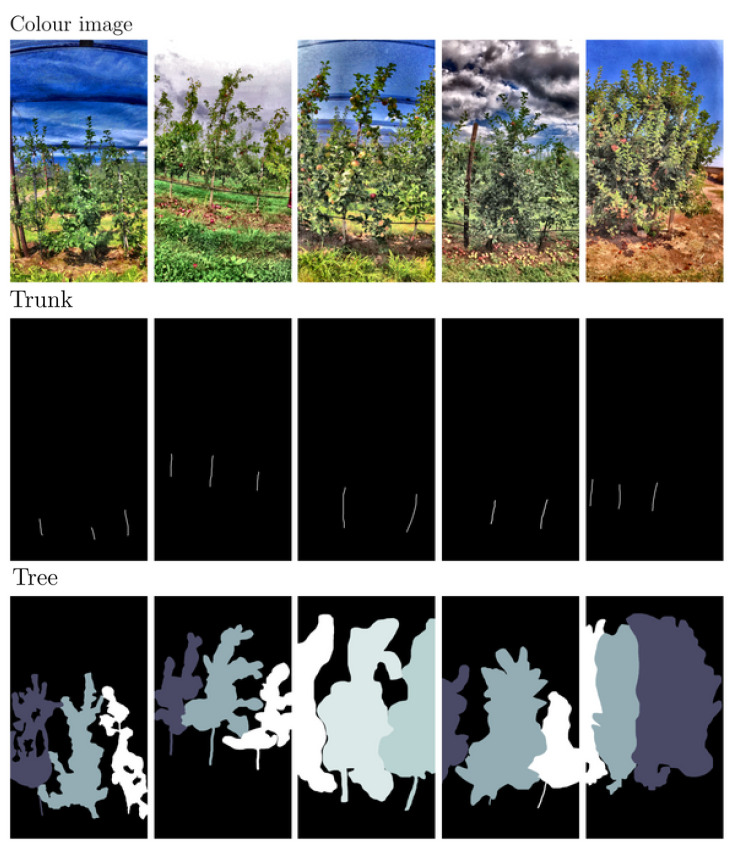
Sample of the data with annotation from apple REFPOP dataset in Table 1. First row stands for the input color images, middle row stands for semantic segmentation ground truth of trunks, and last row stands for instance segmentation ground trunk of trees.

The images were acquired in August 2024 using an Insta360+4K camera (Arashi Vision Inc., Shenzhen, China) with a resolution of 3840×2160 pixels. Some trees were already harvested, and others were harvested later. Based on recorded videos, samples were manually extracted by selecting single frames where a tree appears centered in the image. The final dataset consists of multiple images of each tree taken along the row. The apple REFPOP dataset was constructed using 1375 images. Individual trees were manually annotated, including shoots and trunks. The images include some variability related to the distance between the camera and the trees, light conditions, and camera orientation (as shown in Figure 2). These images are manually labeled for tree segmentation and trunk detection. Annotation activity is performed by four human annotators using Napari software (version 0.5.6). Trunk labeling corresponds to a semantic annotation for all trunks in the first row of trees in front of the camera. Trees located nearest to the camera are labeled with instance-level annotations. The dataset is provided as Supplementary Material to this article.

In addition to our dataset, we tested our method on the dataset of La et al. [13] which contains single trees centered on the images. In this dataset, neighboring foliage trees were visible without their trunks. Table 1 lists the total number of trees in the images of the datasets used in this study.

### 2.2. Trunk Detection

Our objective is to localize the position of each tree by detecting its trunk. Trunk detection step serves as a preprocessing stage for defining the point prompts used in the subsequent tree segmentation task. In this first stage, we investigated the possibility of detecting trunks via instance segmentation and object detection methods. We examined the existing literature on trunk detection. Detection model based on color images of dense orchards were developed by [29,30,31]. Zhao et al. [29] trained version 5 of the small model of You Only Look Once (YOLOv5s) to detect four phenotypes, including trunks and whole trees, while Sun et al. [30] trained version 2 of Segmenting Objects by Locations (SOLOv2) to segment the main trunk from the position of the grafting point. Sapkota et al. [31] detected and segmented tree trunks and branches from color images using trained YOLOv8 and Mask R-CNN models. Their results demonstrated that the detection models can produce trunk labeling in the foreground without the need for depth information. However, their models were adapted for images that capture fragmented views of branches or trunk segments rather than a complete tree. Because the detection models presented in [29,30,31] are not publicly accessible, we had to develop a model. Please note that this is not the core innovation part of our work.

To develop a trunk detection model, we explored two distinct approaches: supervised methods and zero-shot inference. Supervised approaches demonstrated significant success in plant-related applications [32,33,34]. For comparison, we trained one semantic segmentation model and two detection models: a Feature Pyramid Network (FPN) [35], YOLOv8 [36], and YOLOv11 [37]. YOLOv8 and YOLOv11 were fine-tuned using pre-trained MS COCO weights via the publicly available code of Ultralytics (https://github.com/ultralytics/ultralytics (accessed on 19 May 2025)). The FPN model was fine-tuned using weights pre-trained on INRAE images collected at the apple REFPOP site in France in October 2023. The training dataset, as detailed in Table 2, consisted of 55 images selected from five different rows at each apple REFPOP orchard site. It was divided into five folds to assess the overall performance of the models. We applied data augmentation techniques on the training set, such as small rotations (±10°), contrast enhancement, vertical flipping, and zooming, to increase its robustness to variability in viewpoint, tree distance, and lighting. In addition, all images in the training, validation and test sets of the REFPOP dataset were preprocessed using the Contrast Limited Adaptive Histogram Equalization algorithm (CLAHE) [38] to improve local contrast. Model training was conducted on an NVIDIA DGX system with four Tesla V100 GPUs. We used the Adam optimizer with a batch size of 16, employing Distribution Focal Loss for YOLO architectures, and weighted binary cross-entropy for FPN. Early stopping and model checkpoint strategies were applied to prevent overfitting and reduce computational load.

We also compared the supervised trunk detection methods with zero-shot approaches for trunk segmentation. These zero-shot-based approaches leverage a combination of a visual language model (VLM) and a visual foundation model (VFM) to perform inference without task-specific training. In our setup, we used the multimodal open language model (MOLMO) [39] and Grounding Distillation with No Labels (Grounding DINO) [5] to generate point coordinates from textual prompts. Following the general prompting strategy proposed in [40], we used the prompt “detect trunk in middle of image” in a few-shot setting. The generated points were subsequently used as prompt inputs for the Segment Anything Model (SAM), which returned the final trunk segmentation masks. At the final stage of trunk model detection, the Density-based spatial clustering of applications with noise algorithm (DBSCAN) was applied to individually classify each trunk label. A summary of the different model configurations used for trunk detection is presented in Table 3.

### 2.3. Prompt Engineering

Using the detected trunks (Section 2.2), prompts were defined over the apple tree foliage. This strategy involves positioning prompts in specific regions, informed by the spatial configuration of apple trees. Various prompt engineering techniques have been proposed in the literature, each tailored to specific applications. In the medical field, Chen et al. [44] engineered prompts from five images and subsequently generated five masks on new images, selecting the final output mask by a majority vote. The core of their approach is image registration between the new images and the five prompt images. In another study, Wang et al. [45] employed pseudo-label masks generated from a teacher–assistant model to fill gaps in the fine spatial structure of masks produced by the Segment anything model. Similarly, we tailored our own prompts based on the spatial characteristics of the apple trees. Given the substantial variability in tree width across different genotypes [46,47,48,49], and the lack of prior information about tree shape from the trunk alone, point-based prompts were preferred over bounding boxes. Bounding boxes require accurate size estimation, which is often unavailable or inconsistent [50].

**Figure 3:**
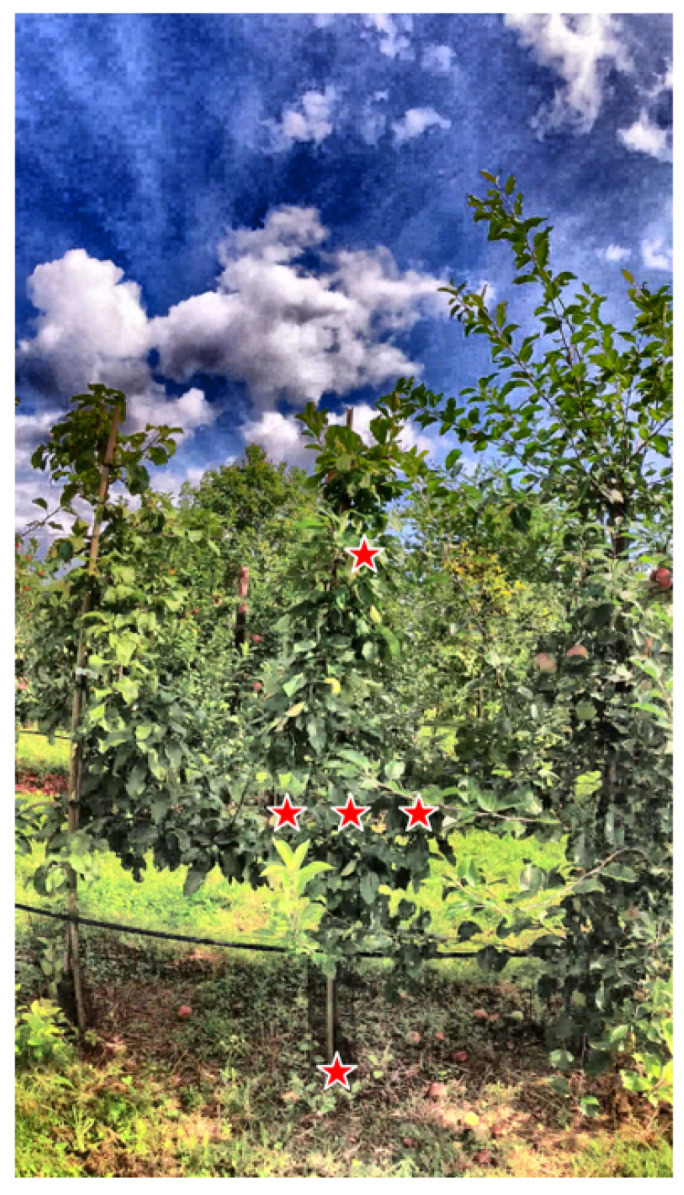
Illustration of points configuration in red star for tree foliage in orchard on a sample of apple REFPOP dataset.

Recall that from each detected trunk, a linear regression line was computed to represent the vertical axis of the tree. Based on this knowledge, prompt points were placed along this axis, from the top of the trunk to the estimated summit of the tree. The tree summit was estimated using two approaches. As a supervised approach, a human operator supplied an approximate tree height in pixel units. As an unsupervised approach, the summit was located automatically from the foreground-segmentation mask produced by the segmentation tool FrontVeg plugin available at Napari (https://www.napari-hub.org/plugins/frontveg (accessed on 10 July 2025)). In both prompt methods, five points are positioned within the foliage region: one at the bottom of the trunk, one at the estimated tree summit, and one at the center of the foliage (midpoint along the regression line). Additionally, two lateral points from the center of the foliage are added at 20% left and 20% right between the regression axis and a border. This border is defined as either the midpoint of the image edge or the neighboring trunk. Figure 3 illustrates a configuration of points. This spatial distribution helps constrain segmentation to the relevant foliage area and reduces the risk of incomplete segmentation. The final coordinates were provided as input prompts to the segmentation model. The process is summarized in detail in the Algorithm 1.

In addition to the supervised and unsupervised prompt strategies described above, we explored zero-shot prompting using two grounding models: MOLMO [39], which generates point-based prompts from textual queries, and Grounding DINO [5], which outputs bounding boxes. Prompt generation used a few-shot prompting formulation with the instruction “detect tree foliage in center”. As demonstrated in [40], incorporating contextual cues and clearly specifying the target object leads to more accurate spatial localization of the desired structures. Table 4 presents a summary of the methods used for prompt generation.
**Algorithm 1** Prompt engineering.**Input:** - *A*: Instance mask of *k* labeled trunks - $mode\in \left\{supervised,unsupervised\right\}$ - **if** mode = supervised: -     h: approximate height of tree (pixels) - **else** -     G: RGB image of mask A
**Output:** Z: Dictionary of *k* tuples (B,T,C,R,L)
1. Determine centroid of each of k labelled trunks2. Identify horizontal neighbors $\left(x_{bl},x_{br}\right)$ for each trunk centroid3. Compute regression line for each trunk and obtain mean angle α4. **for** i = 1 **to** k **do**5.  $A_{i}$ ← mask of trunk i from A6.  $l_{i}$ ← regression line of Ai at angle α7.  $B=(x_{b},y_{b})$ ← bottom endpoint of $l_{i}$8.  **if** mode = supervised **then**9.   $T=(x_{t},y_{t})$ ← point at distance h pixels from B along $l_{i}$10.   **else**11.   *F* ← foreground vegetation mask from image G (FrontVeg plugin)12.   $f_{i}$ ← intersection of $l_{i}$ with mask F13.   $T=(x_{t},y_{t})$ ← top endpoint of the continuous $f_{i}$ segment starting from B14.   **end if**15.   $C=(x_{c},y_{c})$ ← midpoint between B and T.16.   *R* ← $\left(x_{c}+0.2\left(x_{br}-x_{c}\right),y_{c}\right)$17.   *L* ← $\left(x_{c}-0.2\left(x_{c}-x_{bl}\right),y_{c}\right)$18.   Z[i] ← (B,T,C,R,L)19. **end for**20. return Z


### 2.4. Tree Segmentation

Supervised learning has recently made significant progress in instance segmentation. Recent models have been trained for tree segmentation [13,29], leaf segmentation [51,52,53], and fruit segmentation [54,55,56,57], and fruit detection [58]. Apple tree segmentation has been studied by La et al. [13]. As their model was not publicly available, we followed their experimental settings to design a similar model.

In addition, leaf and fruit segmentation has been studied using visual foundation models [59,60,61], that is, a model that has generalization capabilities without the need to be retrained on a specific targeted application domain. In our work, we employed the Segment Anything Model (SAM) [4] for tree segmentation. Released by Meta AI, SAM introduced a novel paradigm in image segmentation by leveraging prompt-based interactions to refine segmentation results on previously unseen images. Unlike traditional segmentation architectures, SAM is composed of three distinct modules: an image encoder, a prompt encoder, and a mask decoder. The image encoder divides the input image into patches and extracts the deep visual features. The prompt encoder processes user-provided prompts (e.g., points and boxes) into a latent embedding. These embeddings are then fused with the image features and passed to the mask decoder, which outputs the segmentation masks.

Although SAM has shown generic capabilities, it may require further refinement. For instance, Ma et al. [62] retrained the initial Segment Anything Model by using a large dataset of typical medical images to gain robustness in low-contrast regions or boundaries that are not clearly visible. Nevertheless, their approach required substantial computational resources such as 20 A100 GPUs for training and one million images. An alternative method for refining the model is to focus on prompts. Torres-Lomas et al. [26] used an automatic mask generator on a pre-trained model to detect grapes in indoor environments, effectively leveraging prompt engineering and visual foundation models for a specific task. Their approach relies on clearly visible grapes, whereas our images were acquired under complex environmental conditions. To address these challenges, we leverage both the RGB images and the point-based prompts defined in Section 2.3, and adopt the Segment Anything Model (SAM) [4,43] as the core segmentation framework for apple trees.

Many SAM variants and extensions have been proposed to further improve performance, accuracy, and speed [43,63,64,65]. In our study, we employed a pre-trained SAM and evaluated its zero-shot segmentation capabilities on apple tree images. We also assessed several recent extensions of SAM, including the version 1 (SAM1) [4] and 2 (SAM2) [43] of Segment Anything, the version 2 of Segment Anything in High Quality (SAM-HQ2) [63] for high-resolution refinement, Fast Segment Anything (FastSAM) [64] for real-time inference, and Robust Segment Anything (RobustSAM) [65], which was designed to improve segmentation quality under complex conditions. Detailed information regarding the models employed in this pipeline is provided in Table 5.

### 2.5. Metrics

We evaluate the quality of trunk detection and tree segmentation by comparing the model predictions with the human-annotated ground truth. For trunk detection, we employ the Average Precision (AP) metric, which measures the precision averaged across multiple recall thresholds. AP quantifies the accuracy of the predicted bounding boxes or masks that are assigned to positive instances and are a standard metric in object detection tasks. A predicted object is considered a true positive if its Dice coefficient with the ground-truth object exceeds a threshold of 0.1. This relatively low threshold reflects our focus on detecting the presence of the trunk, rather than achieving a perfect segmentation match with the ground truth. The detection of the trunk directly influences the quality of the tree-level segmentation that follows in our pipeline.

To assess segmentation quality (for both trunk segmentation and tree segmentation), we use the Dice–Sørensen coefficient (DSC), also known as the Dice score, as recommended in [66]. This metric compares the overlap between predicted and ground truth segmentation masks

$$DSC\left(A,B\right)=2\frac{\left|A\cap B\right|}{\left|A|+|B\right|}$$

where *A* is the set of pixels labeled by the human annotator, and *B* is the set predicted by the model. The Dice score ranges from 0 (no overlap) to 1 (perfect overlap).

To evaluate the systematic under- or over-segmentation, we compute the mean error (ME) for each image:

$$ME=\frac{1}{N}\sum_{i=1}^{N}\left(b_{i}-a_{i}\right)$$

where $a_{i}$ and $b_{i}$ denote the ground-truth and predicted pixel labels, respectively, and *N* is the total number of pixels in the image. Unlike the mean absolute error, the ME retains the sign of the error: a positive ME indicates overestimation of the segmented area, whereas a negative ME indicates underestimation.

Finally, we report precision and recall as complementary metrics

$$\mathrm{Precision}=\frac{TP}{TP+FP}$$

$$\mathrm{Recall}=\frac{TP}{TP+FN}$$

where TP represents the number of true positives, FP is the number of false positives, and FN is the number of false negatives.

## 3. Statistical Analysis

We analyzed the performance of the trunk detection and tree segmentation models using the paired Student *t*-test [67]. We measured the performance of the best model in terms of the mean compared to the others. Because the data used to evaluate each model’s performance are drawn from the same dataset, a paired Student *t*-test is appropriate. Supposing the samples follow normal distribution with varying standard deviation, the paired Student *t*-test was adapted to analyze whether the difference between the mean of best model and the other models is statistically significant.

## 4. Results

In this section, we present the results of the two main components of the tree segmentation pipeline as applied to the datasets described in Section 2. First, we evaluated the performance of the trunk detection part by considering both the segmentation and detection metrics. Second, we assessed the performance of the complete pipeline for individual-tree segmentation on images where trunks are clearly visible in order to ensure reliable performance assessment.

### 4.1. Trunk Segmentation and Detection

**Figure 4:**
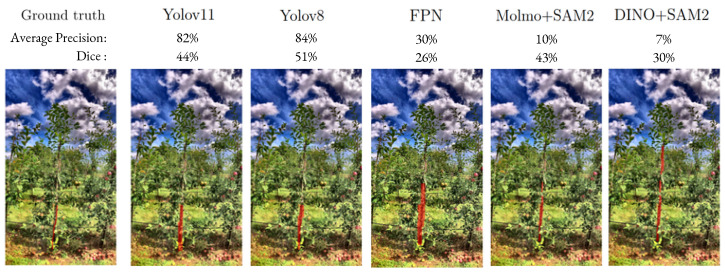
Comparison of trunk segmentation with different models on a sample of the apple REFPOP test dataset. Average precision and Dice scores on the image are also provided.

As shown in Table 6, the trunk segmentation performance remained below 55% across all the tested methods. From a segmentation perspective, the models tend to misestimate the actual geometry of tree trunks as illustrated in Figure 4. However, trunk detection performance, which is our real target, remains satisfactory. Table 7 presents the object detection results, where YOLOv11 achieved significantly the best performance over other models, reaching an Average Precision (AP) of 97% for both the apple REFPOP and La et al. datasets. Furthermore, while not shown in Table 7, YOLOv11 achieved on apple REFPOP, on average, a precision of 71.2% and a recall of 93.2%. In particular, on 138 images on the test set of apple REFPOP where the trunks are clearly visible, 9.66 trunks were missed and 128.34 trunks were correctly detected on average by the YOLOv11 model. High precision indicates a low false positive rate (most predicted trunks are true trunks), whereas high recall indicates few missed actual trunks.

Based on the trunk detection results in Table 7, we selected YOLOv11 as the model perform stage 1 of our pipeline, as it clearly appears to be the best method among the various approaches tested. The results reported in the rest of the study were obtained with YOLOv11 as the trunk detection method.

### 4.2. Tree Segmentation

Table 8 reports the mean tree segmentation performance of the proposed pipeline on each dataset. For both apple REFPOP and the dataset of La et al. [13], the results show that SAMHQ2 consistently outperforms other “Segment Anything” variants. The presence of the High-Quality Output Token in SAMHQ2 is particularly advantageous for segmentation tasks conducted in visually complex environments. The performance of tree segmentation is better for the dataset of La et al. [13]. This is not surprising, as in this dataset the trees are isolated and not intertwined with their neighbors. On the test dataset of La et al. [13], the performance of SAMHQ2 is competitive with the YOLOv8 method trained with the training dataset of La et al. [13]. Moreover, statistical analysis revealed no significant difference in the performance of YOLOv8 compared to SAMHQ2 on this dataset. Furthermore, on the apple REFPOP dataset, the zero-shot SAMHQ2 performs better than the supervised YOLOv8 model. This clearly demonstrates the value of our approach with foundation models in comparison to the supervised method.

**Figure 5:**
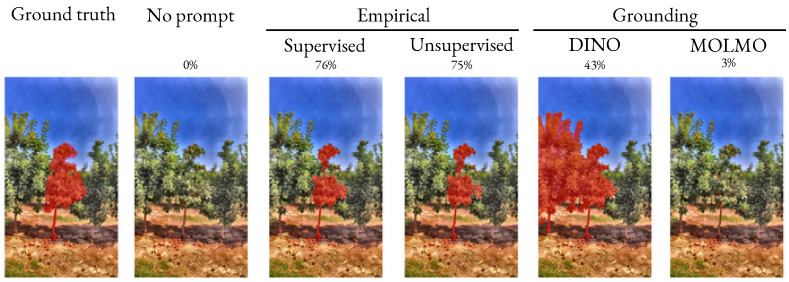
Comparison of tree segmentation generated by SAMHQ2 applied on a sample image of the apple REFPOP test dataset. The Dice scores on the image are also provided.

Because the effectiveness of segmentation depends on the choice of prompt, we analyzed different prompt strategies. As shown in Table 9, explicitly defining prompts results in the highest segmentation accuracy. Indeed, Figure 5 reveals that in the absence of any prompt, models tend to segment easily distinguishable objects rather than the target tree. This finding underscores that tree-focused prompt definitions provide vital contextual cues, enabling models to isolate the intended object within complex scenes. Moreover, the supervised-unsupervised prompting approach outperforms by 13% on average the grounding approaches (based on DINO and MOLMO) for tree segmentation. This can be attributed to the zero-shot paradigm: owing to the lack of domain specialization in tree recognition, foliage features are often indistinguishable for vision foundation models not trained for this specific task. The best prompt approach is an unsupervised approach, in which the height of the tree is automatically estimated. The unsupervised approach significantly outperforms other prompt approaches for SAMHQ2 and RobustSAM.

The diversity within the test dataset originates from the different locations at which the images were captured. In Table 10, we evaluate the segmentation performance for each site using our best model (SAMHQ2 and unsupervised prompt) for each site. We observe that the highest segmentation performance was achieved on the images taken in South Korea [13]. For the apple REFPOP dataset, on the images from France, Belgium and Italy, the performance is in the range of 72–76%, which is 10% higher than on those from Switzerland and Spain. These differences could be due to the foliage density and spacing between trees, which are smaller for Switzerland and Spain, making segmentation more challenging compared to the rest of the apple REFPOP sites. This is in agreement with the high performance obtained for the South Korean dataset, where the trees are almost isolated.

**Figure 6:**
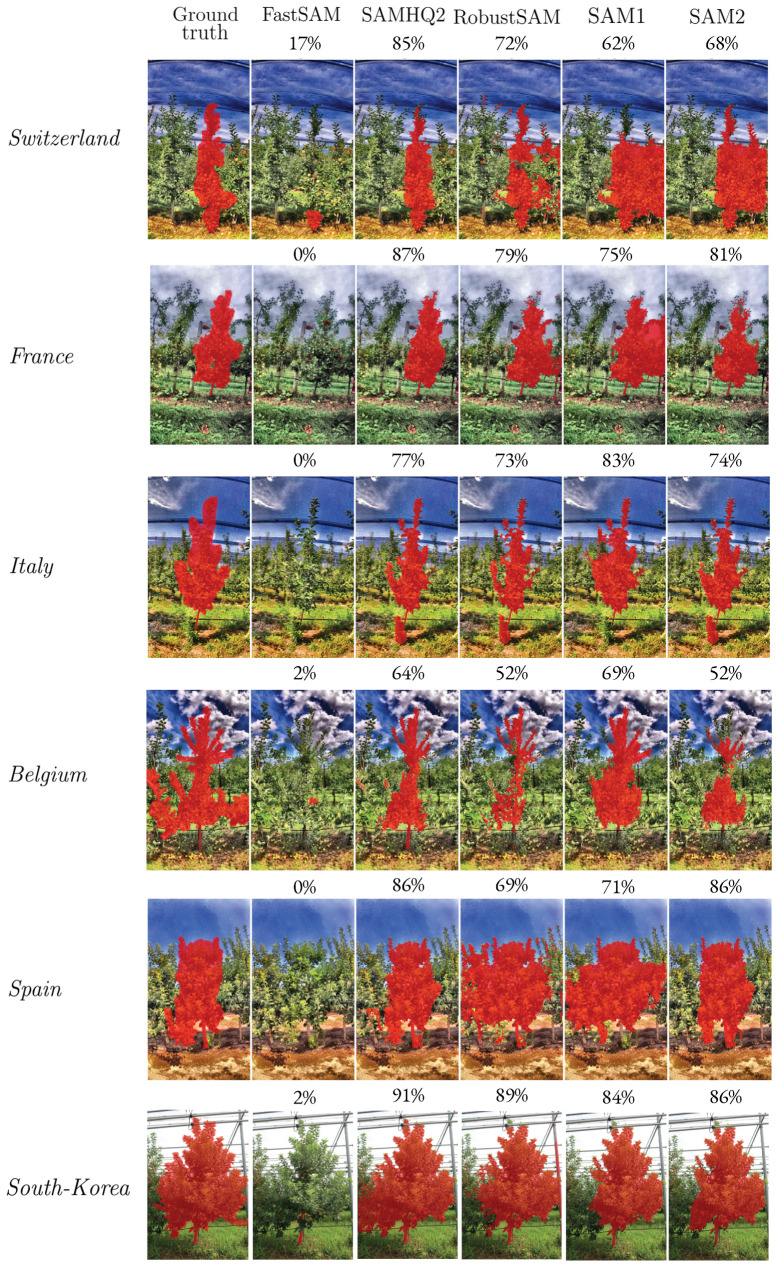
Comparison of tree segmentation accross models and test set locations. The Dice scores on the images are also provided.

Overall, the mean error of the segmentation in Table 10 indicates that the proposed method produces a slight underestimation. This can be explained by the way we establish the ground truth in a rather raw way to limit the annotation time. As shown in Figure 6, we include locally the ground truth of the convex hull of the branches, whereas the segmentation produced by our algorithm better fits the actual contours and provides a more chiseled result, that is, a more accurate result.

## 5. Discussion

In this paper, we presented a method for apple tree segmentation in complex environments, based on a supervised trunk segmentation model for tree detection followed by a zero-shot model for tree segmentation. The proposed pipeline demonstrated good performance, which is comparable to the current supervised state-of-the-art for segmenting apple trees, while operating mostly in a zero-shot learning mode. For each step of our pipeline, we compared various standard methods. In this section, we discuss the directions for possible further improvements to this segmentation pipeline based on the results recorded so far.

The first step in our pipeline is trunk detection. This is the only step in which we select a purely supervised approach. The adaptation of this supervised model to unseen orchards in a robust manner is an important challenge. Furthermore, the task of trunk detection is not limited to applications in orchards; it is also essential for analyzing vineyards, other fruit trees, or forest trees. Therefore, an interesting approach could be to build a generic trunk detector. The model we provided in this study together with the annotated dataset could serve this generic purpose. The current limitation of our detection model is that the woody parts of some of the trunks are hidden by foliage. The labels in these images are smaller compared to those associated with clearly visible trunks. Visibility depends on the tree’s foliage density which may cause occlusion of the trunk, grass density, and the camera’s viewing angle. Therefore, the model is not complex enough to capture these variations. Including more of such images (which were a small minority in our case) may help to reach robustness to occlusion by the foliage. Alternatively, if the camera is equipped with a GPS antenna, the GPS position of the tree could also be used to prompt the images even when the trunk is hardly or not visible.

Regarding the prompt engineering step, some segmentation errors were found in trees with an asymmetric morphology. This issue comes from the symmetric layout of the prompt, which is diamond shaped. If trees are not symmetric, the diamond-shaped prompt will force the model to fuse parts which do not belong to the same tree. A revision of this alternative prompt would involve using a depth-estimated map (such as the one presented in the Supplementary Material) to highlight the presence of some branches oriented towards the camera. Points are then placed on the branches closest to the camera in such a depth map. Only two points are retained: the point farthest to the left of the tree’s center and the point farthest to the right. This could help to design a more individualized prompt for each tree to refine the results obtained in this study.

FastSAM performs poorly compared to other models. The use of FastSAM in our study was solely intended to assess the segmentation capability of a distilled model proposed in the literature, specifically within the context of agricultural scenes. This evaluation is particularly relevant when considering future deployment on embedded systems. The limited performance can be attributed to the training dataset: FastSAM was trained on a small subset of SA-1B, which generally lacks natural images. A promising alternative would be to retrain lightweight models such as FastSAM on a dataset composed of images collected specifically in orchard environments. Another alternative for zero-shot model improvement could be exploring other foundation segmentation models such as Florence 2 [68] or SEEM [69].

Individual tree segmentation remains challenged by trunk occlusion, especially in dense orchards. Zhang et al. [70] addressed similar issues in detecting bagged pears by combining multimodal feature fusion with a cost-sensitive loss function, improving detection under partial visibility. Although their work focuses on fruits, the proposed strategy is transferable to tree segmentation, particularly for handling overlapping structures. Also, for strong intertwined branches, the segmentation remains challenging. One could think of acquiring a view of the trees during winter when there is no foliage as in [9] to disentangle intertwined branches.

## 6. Conclusions

We propose a prompt engineering pipeline that leverages a foundation model to address the challenging task of individual apple tree segmentation in dense orchard environments using only monocular color images. The overall performance of the proposed method demonstrated competitive results in comparison with fully supervised approaches, particularly when considering the zero-shot nature of the tree segmentation step. The method was tested on two datasets. Our trunk detector achieved a successful detection rate of 97% using a trained YOLOv11 model. Our zero-shot tree segmentation method—guided by a prompt placed above the detected trunk—reached a Dice score of 70% without training on the REFPOP dataset and 86% without training on the publicly available benchmark dataset. These results demonstrate that our approach matches or even surpasses fully supervised segmentation methods and unprompted foundation models. Consequently, our findings suggest that the use of LIDAR or depth information is not strictly required to achieve the accurate instance segmentation of foreground tree rows. This opens the door to lightweight, scalable, and cost-effective solutions for orchard monitoring and analysis based solely on visual data and foundation models, guided by well-designed prompts. Future work will aim to further enhance the segmentation robustness and generalization, particularly in cases of occlusions and varying tree morphologies.

## Figures and Tables

**Table 1:** Dataset constructed in this study in comparison with the most related work [13].

Dataset	Images	Trees	Segmentation	Location
REFPOP	275	697	Instance	France
	275	944	Instance	Spain
	275	543	Instance	Italy
	275	841	Instance	Switzerland
	275	809	Instance	Belgium
La et al. [13]	150	150	Semantic	South-Korea

**Table 2:** Training–validation–test split composition for trunk detection and tree segmentation.

Split	Dataset	Location	Images	Total
		France	55	
		Spain	55	
Train	REFPOP	Italy	55	275
		Switzerland	55	
		Belgium	55	
		France	30	
		Spain	30	
Validation	REFPOP	Italy	30	150
		Switzerland	30	
		Belgium	30	
		France	30	
		Spain	30	
	REFPOP	Italy	30	150
Test		Switzerland	30	
		Belgium	30	
	La et al. [13]	South-Korea	30	30

**Table 3:** Models for trunk detection in details.

	Arch.	Param.	Training Set
*Supervised*			
YOLOv11 [37]	CSPDarknet53	2.67M	MS COCO [41]
YOLOv8 [36]	CSPDarknet53	5.35M	MS COCO [41]
FPN [35]	VGG16	1.58M	INRAe [42]
*Zero-shot*			
MOLMO [39]	Molmo 7B+ViT-L	8.2B+639M	PixMo
+ SAM2 [43]			
DINO [5]	SwinT+ViT-L	310M+639M	Grounding-20M
+ SAM2 [43]			

**Table 4:** Summary of method for prompt generation.

Approach	Method	Arch.	Param.	Training Set
Empirical	Supervised prompt	.	.	.
	Unsupervised	.	.	.
	prompt			
Grounding	Molmo [39]	Molmo 7B	8.2B	PixMo
	DINO [5]	SwinT	310M	Grounding-20M

**Table 5:** Models for tree segmentation in details.

	Arch.	Param.	Training Set
*Zero-shot*			
SAM1 [4]	ViT-B	93.7 M	SA-1B
SAM2 [43]	ViT-B	80.8 M	SA-V
SAMHQ2 [63]	ViT-L	224 M	HQSeg-44K
FastSAM [64]	FastSAM-x	68 M	SA-1B
RobustSAM [65]	ViT-B	153 M	LVIS, MSRA10K
			ThinObject-5k
*Supervised*			
YOLOv8 [13]	CSPDarknet53	71.75M	[13]

**Table 6:** Trunk segmentation performance using Dice metric on the test set. The arrow ↓ indicates the set of models used and → indicates dataset used for performance assessment. For each dataset, the figures in bold shows the best model in comparison of figures for each model. The paired student *t*-test was performed for the test results. Scores marked with * indicates statistically significant superiority of the best model in bold over the others (*p*-value < 0.05) on each dataset.

Dataset (→)	REFPOP	La et al. [13]
Models (↓)		
YOLOv11	0.39±0.06 *	0.29±0.05 *
YOLOv8	0.38±0.06 *	**0.53 ± 0.09**
FPN	**0.55 ± 0.05**	0.21±0.10 *
Molmo+SAM2	0.26±0.06 *	0.01±0.01 *
DINO+SAM2	0.01±0.00 *	0.02±0.01 *

**Table 7:** Performance of detection of trunk in the center of image using the AP metric on the test set. The arrow ↓ indicates the set of models used and → indicates dataset used for performance assessment. For each dataset, the figures in bold shows the best model in comparison of figures for each model. The paired student *t*-test was performed for the test results. Scores marked with * indicate the statistically significant superiority of YOLOv11 over the others (*p*-value < 0.05) on each dataset.

Dataset (→)	REFPOP	La et al. [13]
Models (↓)		
YOLOv11	**0.97 ± 0.03**	**0.97 ± 0.05**
YOLOv8	0.91±0.05 *	0.63±0.10 *
FPN	0.74±0.03 *	0.53±0.18 *
Molmo+SAM2	0.42±0.04 *	0.29±0.20 *
DINO+SAM2	0.39±0.06 *	0.60±0.09 *

**Table 8:** Tree segmentation performance based on Dice metric on test datasets. For the zero-shot models in the table, unsupervised prompts were used. The arrow ↓ indicates the set of models used and → indicates dataset used for performance assessment. For each dataset, the figures in bold shows the best model in comparison of figures for each model. The paired student *t*-test was performed for the test results. Scores marked with * indicate the statistically significant superiority of SAMHQ2 over the others (*p*-value < 0.05) on each dataset.

Dataset (→)	REFPOP	La et al. [13]
Models (↓)		
*Zero-shot*		
SAM1	0.64±0.02 *	0.83±0.02 *
SAM2	0.66±0.03 *	0.81±0.03 *
SAMHQ2	**0.70 ± 0.03**	**0.84 ± 0.03**
FastSAM	0.09±0.04 *	0.20±0.15 *
RobustSAM	0.63±0.04 *	0.80±0.04 *
*Supervised*		
YOLOv8 [13]	0.47±0.07 *	0.84±0.04
with La et al.		
YOLOv8 [13]	0.50±0.09 *	0.61±0.08 *
with REFPOP		

**Table 9:** Comparison of prompt strategies on tree segmentation performance using Dice metric applied on apple REFPOP test set. For each model, the figures in bold shows the best approach in comparison of figures for each approach. The paired student *t*-test was performed for the test results. Scores marked with * indicate the statistically significant superiority of the best model in bold over the others (*p*-value < 0.05) on each dataset.

Approach	SAM1	SAM2	SAMHQ2	FastSAM	RobustSAM
No prompt	0.01±0.01 *	0.01±0.00 *	0.00±0.00 *	0.01±0.00 *	0.00±0.00 *
$\begin{array}{c} \mathrm{Empirical}\\ \mathrm{Supervised} \end{array}$	**0.66 ± 0.02**	0.64±0.04	0.68±0.03 *	0.10±0.05	0.62±0.04 *
$\begin{array}{c} \mathrm{Empirical}\\ \mathrm{Unsupervised} \end{array}$	0.64±0.02 *	**0.65 ± 0.03**	**0.70 ± 0.03**	0.09±0.04 *	**0.63 ± 0.04**
$\begin{array}{c} \mathrm{Grounding}\\ \mathrm{DINO} \end{array}$	0.52±0.01 *	0.57±0.02 *	0.55±0.04 *	0.07±0.05 *	0.51±0.02 *
$\begin{array}{c} \mathrm{Grounding}\\ \mathrm{MOLMO} \end{array}$	0.45±0.04 *	0.42±0.03 *	0.19±0.06 *	**0.14 ± 0.13**	0.45±0.01 *

**Table 10:** Tree segmentation performance for each location based on unsupervised prompt using SAMHQ2. The arrow ↓ indicates the set of models used and → indicates dataset used for performance assessment.

Metrics (→)	Dice	Precision	Recall	Mean Error
Location (↓)				
Switzerland	0.60±0.17	0.87±0.12	0.50±0.23	−0.05±0.03
Spain	0.62±0.06	0.48±0.07	0.88±0.04	+0.15±0.06
Italy	0.72±0.09	0.86±0.08	0.66±0.16	−0.06±0.08
France	0.72±0.04	0.71±0.13	0.77±0.08	+0.02±0.04
Belgium	0.76±0.05	0.91±0.02	0.66±0.07	−0.05±0.01
South Korea [13]	0.84±0.03	0.88±0.03	0.80±0.04	−0.04±0.04

## Data Availability

REFPOP dataset: https://doi.org/10.57745/DZBMAM.

## Supplementary Materials

The following supporting information can be downloaded at: https://doi.org/10.57745/DZBMAM, https://www.napari-hub.org/plugins/frontveg (accessed on 10 July 2025).

## Abbreviations

The following abbreviations are used in this manuscript:

LiDAR	Light Detection And Ranging
CLAHE	Contrast Limited Adaptive Histogram Equalization
FPN	Feature Pyramid Network
YOLO	You Only Look Once
VLM	Visual Language Model
VFM	Visual Foundation Model
MOLMO	Multimodal open language model
SAM	Segment Anything Model
SAM1	Segment Anything Model, version 1
SAM2	Segment Anything Model, version 2
SAMHQ2	Segment Anything Model in High Quality, version 2
FastSAM	Fast Segment Anything Model
RobustSAM	Robust Segment Anything Model
DBSCAN	Density-based spatial clustering of applications with noise
DINO	Distillation with No Labels
ViT	Vision transformer
ViT-B	ViT Base
ViT-L	ViT Large
AP	Average precision
DSC	Dice–Sørensen
ME	Mean Error
TP	True positive
FP	False positive
FN	False negative

## Technical Terms

The following terms are used in this manuscript:

RGB cameras or images	Synonym of colour cameras or images while RGB means
	Red, Green and blue
Stereovision techniques	Computer vision methods that use at least two RGB cameras
	from different viewpoints to reconstruct the scene in 3D space.
Prompt	Structured input (text, point, boxes or masks) used to guide
	a vision model on specific objects or regions
Prompt engineering	Methodological process to design and optimize input
	(e.g prompt) to effectively guide a model toward specific output.
Row	A set of trees ordered in line or column
Napari software	Image processing tools for multidimensional data
Instance segmentation	Computer vision task to label individual objects in the same class
Object detection	Computer vision task to draw object around box
YOLOv5s, YOLOv8, YOLOv11	Different versions of YOLO series
SOLOv2	Segmenting Objects by LOcations version 2
Mask R-CNN	Mask Region-based Convolutional Neural Network
Fine-tuning	Computer vision process to train a pre-trained model on
	a smaller or specific dataset.
Training dataset	Data used to fit the model.
Validation dataset	Data used to tune hyperparameters and evaluate performance
	during training.
Test dataset	Data used to assess the model’s final performance on unseen data.
Data augmentation	Computer vision techniques to increase the diversity of data by
	artificially applying transformations to the original data.
CLAHE algorithm	Computer vision techniques to enhance local image contrast
	while limiting noise amplification
Grafting point	Anatomical interface between the scion and rootstock in
	a grafted tree.
Genotype	Individual distinguished based on its specific combination
	of genetic markers or alleles.
Image registration	Computer vision method to align at least two images of the same
	scene taken at different times, viewpoints, or sensors by
	transforming them into a common coordinate system.
Pseudo-label mask	Mask automatically generated by a semi-supervised model
	used as a substitute for ground truth labels.
Teacher–assistant model	An intermediate model that helps transfer knowledge from
	a large teacher model to a smaller student model
	during distillation.
Grouding approach	Approach linking text as a prompt or description to localize
	objects or regions in an image.
Latent embedding	Low-dimensional vector representation at the end of a neural
	network that captures the key features of input data.
